# Multimodality treatment approach in management of primary peripheral primitive neuroectodermal tumor of the orbit

**DOI:** 10.4103/0301-4738.55067

**Published:** 2009

**Authors:** Usha R Kim, Vipul Arora, J Devanand, Hadi M Khazei

**Affiliations:** 1 Orbit, Oculoplasty and Oncology Clinic, Aravind Eye Hospital and Postgraduate Institute of Ophthalmology Madurai, Tamil Nadu, India; 2 Oncologist Apollo Hospital, Madurai, India

**Keywords:** Extraosseous Ewing's sarcoma, nonspecific enolase, primitive neuroectodermal tumor

## Abstract

Primitive neuroectodermal tumor is a small round cell malignancy which rarely involves the orbit. We report a case of a two-year old male child presenting as unilateral eccentric proptosis with extraconal and intraconal mass, diagnosed as primary peripheral primitive neuroectodermal tumor (pPPNET) on histopathology and immunohistochemistry. There is no defined consensus in the management of these tumors due to its rare presentation. We describe its distinguishing features with emphasis on multimodal and aggressive treatment approach which ensures appropriate management of these cases.

Primitive neuroectodermal tumor (PNET) is a broad term that includes a wide array of lesions with varying differentiating potential affecting both the central and peripheral nervous system.[1] These are small round cell tumors of neuroectodermal origin with high malignant potential. Peripheral PNET has been grouped into “Askin” tumor (most common; thoracopulmonary region), pigmented neuroectodermal tumor and Ewing's sarcoma/PNET group (extraosseous Ewing's group).[2] To the best of our knowledge till now only nine cases of orbital involvement have been reported in literature.[3] Herein we report an additional case of this rare entity.

## Case Report

**Figure 1:**
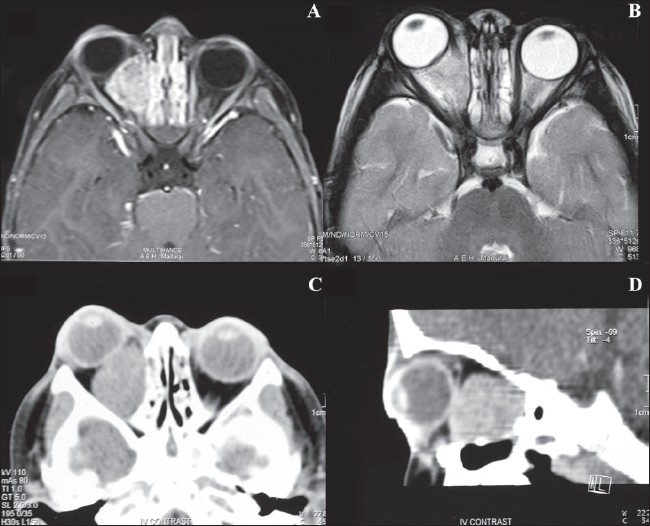
(A and B) MRI (T1 and T2 Wt Scan) suggestive of ovoidshaped mass which is hypointense on T1W and hyperintense in T2W/FLAIR sequences involving inferomedial quadrant of the right orbit. (C and D) CT scan shows increase in size of lesion involving extra and intraconal compartments of the right orbit engulfing the optic nerve sheath complex, medial rectus, inferior rectus and inferior oblique muscles

A two-year-old male child presented with history of progressive prominence of the right eye for the past three months. On external ocular examination there was an upward and lateral deviation of the right eye with eccentric proptosis. The child was not cooperative for visual assessment. Anterior segment examination of the right eye was unremarkable except for a sluggish pupillary response; however, there was no relative apparent pupillary defect. Fundus evaluation of the right eye showed choroidal folds with normal optic disc. The patient had undergone magnetic resonance imaging (MRI) three months earlier, suggestive of an ovoid-shaped, contrast enhancing lesion measuring 2.59 × 2.06 × 1.86 mm, involving the inferomedial quadrant of the right orbit pushing the globe upward and laterally. The lesion was indistinguishable from the inferior rectus, inferior oblique and medial rectus muscles, however, the optic nerve was normal. Left orbit and brain scans were normal [Fig. 1].

Subsequent imaging by computed tomography (CT) scan showed increase in the size of the lesion that involved both extraconal and intraconal compartments of the right orbit. The lesion was now engulfing the optic nerve sheath complex [Fig. 1].

**Figure 2:**
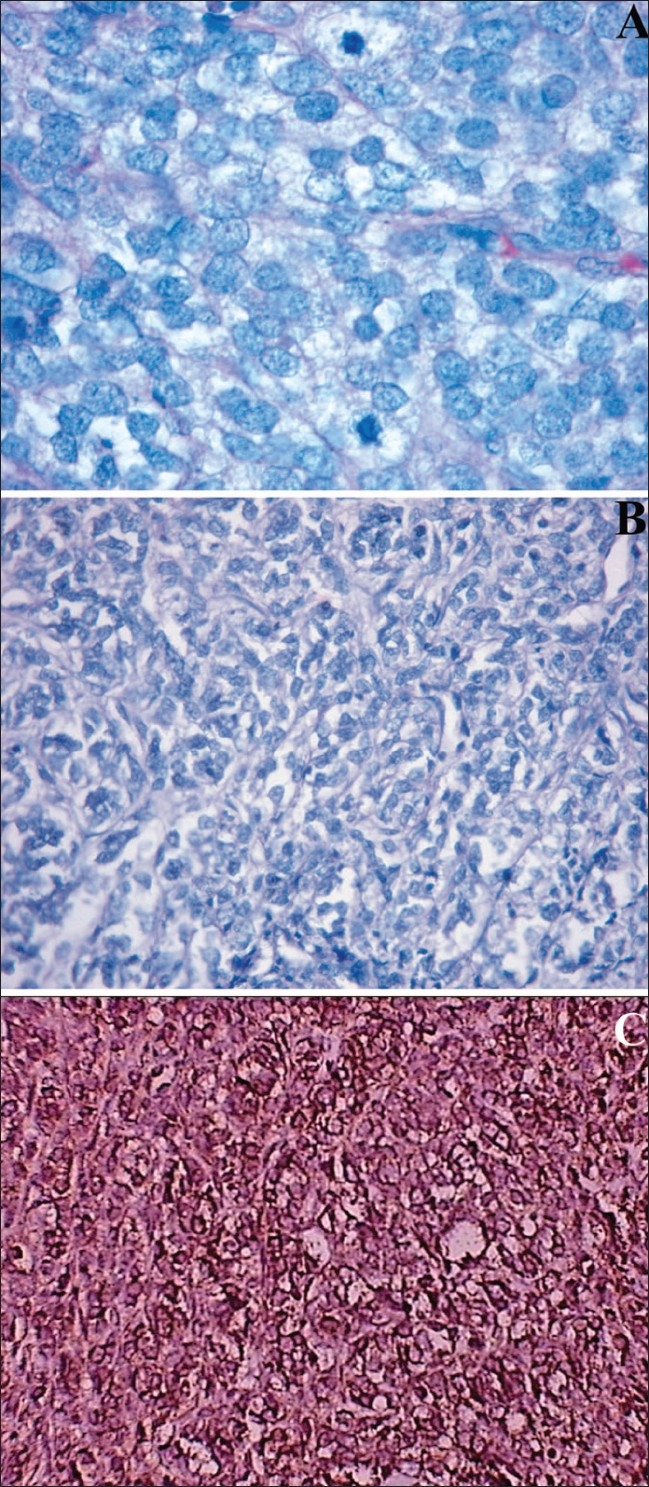
Histopathology of primitive neuroectodermal tumor showing (A) Sheets and lobules of round cells with vesicular nuclei and clear cytoplasm (hematoxylin eosin; 100 X magnification) (B) Showing sheets of uniform round cells with high mitotic activity (H&E, ×40) (C)The tumor cells demonstrate CD99 positivity

An incision biopsy of the mass was done via inferior transconjunctival approach. Paraffin sections were stained with Hematoxylin-Eosin and Periodic Acid-Schiff stains. Histopathologic examination of the tumor revealed diffuse collection of small malignant round to oval cells [Fig. 2]. Cells had vesicular nuclei, inconspicuous nucleoli, scanty cytoplasm and exhibited high mitotic activity (6 MF/HPF) with rosette formation in some areas. The PAS reaction was negative. Sections were studied immunohistochemically for HIC-2 gene (CD99), neuron-specific enolase (NSE), myoglobin, desmin, CD45 and Factor VIII. The majority of cells were positive for HIC-2 gene and NSE. Based on the above findings the patient was diagnosed to have PNET of the right orbit. Extensive systemic investigations were done subsequently to rule out any other foci of tumor, these included complete hemogram, CT thorax, ultrasonography of abdomen and pelvis, bone scan and bone marrow biopsy. These were all normal and therefore diagnosis of primary orbital malignancy was made.

**Figure 3:**
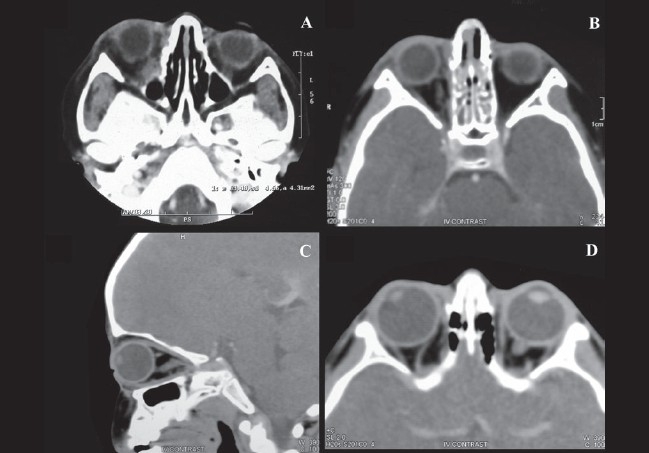
(A) CT scan post chemotherapy suggestive of reduction in mass infero-medially and posteriorly in the right orbital apex. (B) CT scan post radiotherapy and chemotherapy with further reduction of tumor mass. (C and D) CT scan on the last follow-up (30 months) shows no recurrence or residual disease

The child was subsequently treated with 12 cycles of chemotherapy which included six cycles of vincristine, adriamycin and cyclophosamide alternating with six cycles of ifosfamide and etoposide. This was followed with external beam radiotherapy of 44 Gy dose over 22 fractions. Serial orbital imaging was done every four months, showing a gradual reduction in the mass. Last CT scan done at the end of 30 months of follow-up showed no evidence of local recurrence or residual disease [Fig. 3]. As a known side-effect of radiotherapy, the child developed a posterior subcapsular cataract in the right eye. Parents were explained the need for regular follow-up, six-monthly for first two years and then yearly.

## Discussion

PNET is a malignancy which usually peaks in adolescents and young adults with no gender bias.[3] They are classified under Ewing's family of tumors (extraosseous type), with which it shares histopathological and cytogenetic similarity, that is, translocation t(11;22)(q24;q12).[4,5]

Microscopically, primary peripheral PNET is cellular tumor with characteristic small round cells with hyperchromatic nuclei, and a high nuclear–cytoplasmic ratio. Ultrastructural studies by electron microscopy show cytoplasmic filaments and neurosecretory granules.[5] This may aid in the diagnosis of neuroectodermal tumor and to differentiate it from extraosseous Ewing's sarcoma.[2,5] There are varying degrees of neuronal differentiation, beginning with NSE expressivity, followed by Homer-Wright rosette formation, phenotypic ganglion cell differentiation, and finally by neurofilament protein expression.[6] Presence of Homer-Wright rosettes is associated with these tumors but it is not diagnostic of these tumors.[7] Our case was NSE-positive with presence of rosettes.

The differential diagnosis of primary peripheral PNET of orbit includes Ewing's sarcoma, lymphoma, neuroblastoma, hemangioblastoma and small cell osteogenic sarcoma.[6] Immunohistochemistry helps in differentiating this entity from other tumors. Negative PAS reaction rules out Ewing's sarcoma. Lack of staining with desmin and myoglobin excludes the possibility of rhabdomyosarcoma. CD45 helps to differentiate it from lymphoma and Factor VIII to rule out vascular tumors. However, specific immunocytological markers for primary peripheral PNET are HIC-2 gene (CD 99),[8] NSE and synaptophysin and glial fibrillary acidic protein (GFAP).[9] The first two cytological markers were positive in our case, thus confirming the diagnosis of primitive neuroectodermal tumor.

Out of nine previous reported cases of isolated orbital PNET,[2,3,10] age group varied from less than one year to 13 years with two cases reported in adults (52 years and 28 years). There was a predilection for lateral orbit in five cases, inferior orbit in three cases and superior orbit in one case. Bony involvement was present in three cases. In our case, tumor was present initially in the inferior orbit but rapidly involved both intraconal and extraconal compartments, although there was no bony involvement. Management varied as three cases were treated with external beam radiotherapy and two cases with bone involvement were managed by both external beam radiotherapy and chemotherapy. The remaining four patients were not given additional chemotherapy or radiotherapy. We managed our case with 12 cycles of chemotherapy followed by external beam radiotherapy with successful reduction in the mass with no recurrence. Long-term follow-up is mandatory in the management of neuroectodermal tumors as metastasis and recurrence are known in this group. Moreover, as these children are on long-term chemotherapeutic drugs, they should be observed for treatment-related secondary malignancies. Our case has been followed up for 30 months and has no recurrence of the disease.

Primary peripheral PNET of the orbit is a rare entity that poses a diagnostic challenge. Imaging and histopathology though supportive do not confirm the diagnosis. Immunocytology helps in the confirmation of diagnosis. Management should be aggressive using multimodality treatment approach given at the appropriate time. These patients should be followed up for life to rule out recurrence, metastasis and treatment-related malignancies.
